# Analysis of the Modulation of RAF Signaling by 14-3-3 Proteins

**DOI:** 10.1007/s11538-026-01634-2

**Published:** 2026-04-21

**Authors:** Peter Carlip, Edward C. Stites

**Affiliations:** Department of Laboratory Medicine, Yale Cancer Center, Yale School of Medicine, 300 George St., New Haven, CT 06512 USA

## Abstract

The regulation of cellular biochemical signaling reactions includes the modulation of protein activity through a variety of processes. For example, signaling by the RAF kinases, which are key transmitters of extracellular growth signals downstream from the RAS GTPases, is modulated by dimerization, protein conformational changes, post-translational modifications, and protein-protein interactions. 14-3-3 proteins are known to play an important role in RAF signal regulation, and have the ability to stabilize both inactive (monomeric) and active (dimeric) states of RAF. It is poorly understood how these antagonistic roles ultimately modulate RAF signaling. To investigate, we develop a mathematical model of RAF activation with both roles of 14-3-3, perform algebraic and numeric analyses, and compare with available experimental data. We derive the conditions necessary to explain experimental observations that 14-3-3 overexpression activates RAF, and we show that even arbitrarily strong binding of 14-3-3 to RAF dimers alone could not necessarily explain this observation. Our integrated analysis also suggests that RAF–14-3-3 binding is relatively weak (significant amounts of RAF would remain unbound if only the first affinity were a factor), and instead that changing avidity more directly controls the bound fraction. Lastly we consider the limit at which RAF–14-3-3 interactions are driven solely by avidity, which allows for significant simplifications to the interaction model. Overall, our work presents a mathematical model that can serve as a foundational piece for future, extended, studies of signaling reactions involving regulated RAF kinase activity.

## Introduction

Many biological functions are controlled by the outputs of protein networks. For example, cellular proliferation can be regulated by the RAS-RAF-MEK-ERK network. There are three RAF proteins in humans: ARAF, BRAF, and CRAF, which are encoded by *ARAF*, *BRAF*, and *RAF1* genes respectively, as well as the closely related KSR1 and KSR2 proteins which are encoded by *KSR1* and *KSR2*. In response to various stimuli, the RAS proteins (KRAS, NRAS, and HRAS) are activated, and the activation of RAS leads to the activation of RAF which in turn activates MEK to propagate the signal onward. Although this basic concept of a signal propagating down a cascade of reactions is appealing and intuitive, actual signaling is more complex both with respect to the organization of the network and to the steps that influence whether a protein is in an “active” form that can further propagate signals or is in an “inactive” form and cannot. The activation of RAF proteins, in particular, is quite complicated (Lavoie and Therrien 2015).

Although significant progress has been made in determining the steps involved in RAF activation, many aspects of the process remain poorly understood. For example, the modulation of RAF signals by 14-3-3 proteins is well-appreciated to occur but incompletely understood. (There are 7 different 14-3-3 proteins: 14-3-3 $\beta$, $\varepsilon$, $\gamma$, $\eta$, $\sigma$, $\theta$ (or $\tau$ in some sources), and $\zeta$, coded by the genes *YWHAB*, *YWHAE*, *YWHAH*, *YWHAG*, *SFN*, *YWHAQ*, and *YWHAZ* respectively. These isoforms form homodimers and heterodimers in various combinations. For simplicity, we refer to any 14-3-3 dimer as 14-3-3.) It has been shown that 14-3-3 binds RAF at two separate phosphoserine sites: serine 729 (S729) and serine 365 (S365) in BRAF (Simanshu and Morrison 2022), which correspond to serine 621 and serine 259 respectively in CRAF (Morrison et al. 1993). (The BRAF numbering is used throughout). 14-3-3 binding at S365 is inhibitory and binding at S729 is activating (Morrison et al. 1993; Tzivion et al. 1998). This is due to the two roles played by 14-3-3 binding: stabilization of closed, inactive RAF monomers (Park et al. 2019), which involves binding at both S365 and S729, and stabilization of (potentially active) RAF dimers, involving binding solely at S729 of each monomer (Park et al. 2019; Martinez Fiesco et al. 2022). Fundamental aspects of these interactions remain incompletely defined; for example, measurements of the affinities of these bindings have varied widely (Muslin et al. 1996; Tzivion et al. 1998; Ghosh et al. 2015; Zhang et al. 2021), and the degree of avidity, or enhanced binding due to multivalent interactions (Erlendsson and Teilum 2021), remains unclear.

In separate studies, we developed a mathematical model of RAF signaling that includes dimerization, conformational autoinhibition, and the binding of various RAF inhibitor drugs (Mendiratta and Stites 2023), and then used that model to investigate the regulation of RAF signals by 14-3-3 (Mendiratta et al. 2025). That modeling suggested that the stabilization of the conformationally inactive form of RAF by 14-3-3 proteins can amplify the phenomena of “paradoxical activation,” in which RAF inhibitors actually cause increases in RAF signaling (Kholodenko 2015; Rukhlenko et al. 2018; Fröhlich et al. 2023; Mendiratta and Stites 2023). Our interpretation was also tested and supported by experimental results.

In order to create a tractable model of RAF regulation by 14-3-3 and the modulation of RAF inhibitor drug responses, the work in Mendiratta and Stites (2023) and Mendiratta et al. (2025) necessarily simplified many aspects of RAF activation, including details of RAF–14-3-3 interactions. One such simplification was to only consider 14-3-3 binding to RAF monomers or dimers as a single equilibrium reaction between a 14-3-3 complex with both phosphorylated sites on RAF monomer (S365 and S729) or on a RAF dimer (S729 on each protomer in the dimer), rather than modeling the sites independently. It is not clear whether or how this simplification impacts model-based insights into 14-3-3 regulation of RAF.

With 14-3-3 a known, critical, modulator of RAF signals, we desired to study the effects of 14-3-3 on RAF activation without the previously used simplification. We constructed a model of RAF dimerization and the interaction of 14-3-3 with monomeric and dimeric forms of RAF. In order to make this model tractable, we did not include RAF inhibitor binding. Our new model can be solved analytically, allowing us to take derivatives of concentrations with respect to free 14-3-3 concentration. By evaluating values within a reasonable physiological range, we conclude that 14-3-3 binding to RAF is most likely avidity driven. By evaluating the Mendiratta et al. (2025) transfection experiments, we also find that the observation that increased 14-3-3 increases dimerization, which combined with our new modeling suggests that only double-binding of 14-3-3 to RAF is significant. This work further substantiates earlier work, by justifying a critical simplification of Mendiratta et al. (2025), and provides a foundational building-block that can be used to construct extended models of RAS/RAF and/or of growth factor signaling networks.

In Section 2, we describe the system of equilibrium equations that makes up our model, finding an analytical solution for the concentration of RAF in each conformation as a function of free 14-3-3 and total RAF. In Section 3, we use this solution to find numerical values of the dimer concentration given variation of rate constants, suggesting that strong double-binding may be insufficient to explain the observed response of dimer concentration to the addition of 14-3-3, and weak single binding may be necessary. By weak, we mean K comparable 14-3-3 concentration (likely at least hundreds of nanomolar, possibly up to tens of micromolar), implying a significant amount of RAF would be unbound given similar RAF and 14-3-3 concentrations without the effects of double binding. We show this more rigorously in Section 4, given assumptions that single binding interactions resemble one another, as do double binding interactions.

## Model

### Description

In this model of RAF–14-3-3 interactions, RAF can be in one of two conformations: open ($[\textit{Raf}_O]$) and closed ($[\textit{Raf}_C]$). When open, RAF may bind to 14-3-3 at the site S729, producing $[\textit{Raf}_{729O}]$. It may also dimerize when open, whether bound to 14-3-3 at S729 or unbound (though only one protomer may be bound initially), producing $[Dim_1]$ and $[Dim_0]$ respectively. RAF may also bind 14-3-3 when dimerized ($[Dim_0] \rightarrow [Dim_1]$). In addition, when a RAF dimer is bound to 14-3-3 at S729 of one of the component protomers, the second S729 may bind as well ($[Dim_1] \rightarrow [Dim_2]$).

**Fig. 1:**
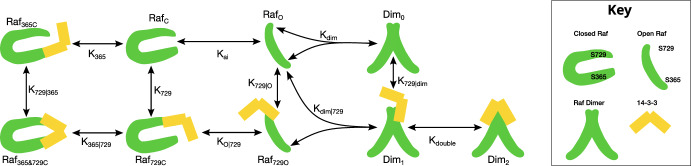
The RAF–14-3-3 model, including conformational changes, dimerization, and independent 14-3-3 binding at multiple RAF sites. The eleven reactions shown reduce to eight equilibrium equations, as each of the three cycles (S729 and S365 attachment to closed monomer, S729 attachment and opening, and S729 attachment and dimerization) allows for a simplification from detailed balance

In the closed conformation, meanwhile, RAF can bind 14-3-3 at either S365 or S729, producing $[\textit{Raf}_{365C}]$ and $[\textit{Raf}_{729C}]$ respectively. When one site is bound, the other may also bind, producing $[\textit{Raf}_{365 \&729C}]$. RAF may close from either $[\textit{Raf}_O]$ or $[\textit{Raf}_{729O}]$, producing $[\textit{Raf}_C]$ and $[\textit{Raf}_{729C}]$ respectively, but may not open when bound at S365. All binding to 14-3-3 in either state requires free 14-3-3 ($[\text {14-3-3}]$). These definitions are listed in Table 1, and the model is also shown graphically in Figure 1.

**Table 1:** Definitions of concentration variables. All concentrations are in molarity, all times are in seconds

$[\textit{Raf}_C]$	[Free Closed RAF]
$[\textit{Raf}_O]$	[Free Open RAF]
$[\textit{Raf}_{365C}]$	[Closed RAF–14-3-3 at S365]
$[\textit{Raf}_{729C}]$	[Closed RAF–14-3-3 at S729]
$[\textit{Raf}_{365\&729C}]$	[Closed RAF–14-3-3 at S365 and S729]
$[\textit{Raf}_{729O}]$	[Open RAF–14-3-3 at S729]
$[Dim_0]$	[Free RAF Dimers]
$[Dim_1]$	[Single-Bound RAF Dimers]
$[Dim_2]$	[Double-Bound RAF Dimers]

The phosphorylation state of the serine residues is not modeled, and other aspects of RAF activation are abstracted into various rate constants (e.g. RAS binding as part of what determines the open:closed balance). This allows for a general, solvable model and accounts for uncertainty in the exact order of some aspects of RAF activation, but also means the model’s rate constants do not always correspond perfectly with the rates of specific chemical reactions. $K_{ai}$, for instance, is not the ratio of the physical closing to opening rates. It expresses the ratio of concentrations in the open and closed states, which is affected by the rate of RAS binding and possibly inhibitory phosphorylation of RAF by ERK.

### Equilibrium Solution

To solve for the equilibrium conditions of our model, we define the equilibrium constants

1 $$\begin{aligned} K_{365}&= \quad \frac{[\textit{Raf}_{365C}]}{[\textit{Raf}_C][\text {14-3-3}]}, \end{aligned}$$

2 $$\begin{aligned} K_{729}&= \quad \frac{[\textit{Raf}_{729C}]}{[\textit{Raf}_C][\text {14-3-3}]}, \end{aligned}$$

3 $$\begin{aligned} K_{729|365}&= \quad \frac{[\textit{Raf}_{365\&729C}]}{[\textit{Raf}_{365C}]}, \end{aligned}$$

4 $$\begin{aligned} K_{365|729}&= \quad \frac{[\textit{Raf}_{365\&729C}]}{[\textit{Raf}_{729C}]}, \end{aligned}$$

5 $$\begin{aligned} K_{ai}&= \quad \frac{[\textit{Raf}_C]}{[\textit{Raf}_O]}, \end{aligned}$$

6 $$\begin{aligned} K_{729|O}&= \quad \frac{[\textit{Raf}_{729O}]}{[\textit{Raf}_O][\text {14-3-3}]}, \end{aligned}$$

7 $$\begin{aligned} K_{O|729}&= \quad \frac{[\textit{Raf}_{729O}]}{[\textit{Raf}_{729C}]}, \end{aligned}$$

8 $$\begin{aligned} K_{dim}&= \quad \frac{[Dim_0]}{{[\textit{Raf}_O]}^2}, \end{aligned}$$

9 $$\begin{aligned} K_{729|dim}\&= \quad \frac{[Dim_1]}{[Dim_0][\text {14-3-3}]}, \end{aligned}$$

10 $$\begin{aligned} K_{dim|729}\&= \quad \frac{[Dim_1]}{[\textit{Raf}_{O}][\textit{Raf}_{729O}]}, \end{aligned}$$

11 $$\begin{aligned} K_{double|dim}&= \quad \frac{[Dim_2]}{[Dim_1]}. \end{aligned}$$

The symbol $|$ is read as given, borrowing notation from conditional probability. It represents the equilibrium of binding at the site listed before the bar, given the condition listed after (14-3-3 binding at a particular site, open RAF monomers, or free RAF dimers). All equilibrium constants are in the form $k_{\text {forward}}/k_{\text {reverse}}$, with the forward direction described above. For all binding interactions, this means they are in $K_A$ form ($k_{on}/k_{\textit{off}}$).

All reactions are reversible, and detailed balance is maintained:

12 $$\begin{aligned} K_{365|729}&= \frac{K_{365}K_{729|365}}{K_{729}}, \end{aligned}$$

13 $$\begin{aligned} K_{dim|729}&= \frac{K_{729|dim}{K_{dim}}}{K_{729|O}}, \end{aligned}$$

14 $$\begin{aligned} K_{O|729}&= \frac{K_{729|O}}{K_{729}K_{ai}}. \end{aligned}$$

This allows us to reduce our system of eleven equations (Equations (1) to (11)) to eight.

To find the equilibrium concentration of each species, we take an expression for the conserved total concentration of RAF as a sum of its states, and replace the individual states with expressions in terms of a single state ($[O]$) using the equilibrium relations above:

15 $$\begin{aligned} \begin{aligned} \text {Raf}_{\text {total}} ={}&[\textit{Raf}_C] + [\textit{Raf}_{365C}] + [\textit{Raf}_{729C}] + {[}\textit{Raf}_{365\&729C}] + [\textit{Raf}_O] + [\textit{Raf}_{729O}] \\&+ 2([Dim_0] + [Dim_1] + [Dim_2]) \end{aligned} \end{aligned}$$

16 $$\begin{aligned} \begin{aligned} ={}&K_{ai} [\textit{Raf}_O] (1 + K_{365}[\text {14-3-3}] + K_{729}[\text {14-3-3}] + K_{729|365} K_{365} [\text {14-3-3}]) \\&+ [\textit{Raf}_O] (1 + [\text {14-3-3}] K_{729|O}) \\&+ 2 {[\textit{Raf}_O]}^2 K_{dim} (1 + K_{729|dim}[\text {14-3-3}] + K_{double|dim} K_{729|dim} [\text {14-3-3}]) \end{aligned} \end{aligned}$$

This system is quadratic in $[\textit{Raf}_O]$, given a constant free 14-3-3, and so it allows a solution for $[\textit{Raf}_O]$ using just the quadratic formula. As total 14-3-3 increases monotonically with free 14-3-3 ($\partial \text {[Total 14-3-3]}/\partial \text {[Free 14-3-3]} > 0$), this is just a rescaled version of the system with constant total RAF and 14-3-3. It is also possible to calculate total 14-3-3 once the system is solved with a given free 14-3-3, and graph the numerical results with respect to total 14-3-3. In addition, dimers always become more common when more RAF is added or affinity of 14-3-3 to dimers ($K_{729|dim}$ or $K_{double|dim}$) is increased and become less common when RAF tends towards closed (increased $K_{ai}$, e.g. because of reduced RAS activation). These derivative results are shown in Online Resource 2.

From this solution, it is possible both to assess the system analytically and to numerically solve it for a given set of concentrations and equilibrium constants. Both are shown below.

**Table 2:** All binding constants above are order-of-magnitude estimates of plausible values for protein-protein binding, rather than measured values. The concentration of RAF was derived from the number of counts found in Kulak et al. (2014) for HeLa cells, counting all isoforms of RAF. They were scaled to the necessary cell volume to give a total RAS concentration matching Fujioka et al. (2006), with the assumption that KRAS (which Kulak did not measure) makes up half of total RAS. 14-3-3 concentrations were determined using the same process, under the assumption that $\sim$1/3, 1/30, and 1/300 of 14-3-3 $\zeta$ and $\epsilon$ were available for binding in the high, medium, and low cases respectively. Those isoforms were chosen based on the finding of Martinez Fiesco et al. (2022) that 75% of 14-3-3 bound to RAF was of those isoforms

Variable	Default Value
$K_{365}$	$10^7$ $\text {M}^{-1}$
$K_{729}$	$10^7$ $\text {M}^{-1}$
$K_{729|365}$	1
$K_{ai}$	1
$K_{729|O}$	$10^7$ $\text {M}^{-1}$
$K_{dim}$	$10^7$ $\text {M}^{-1}$
$K_{729|dim}$	$10^7$ $\text {M}^{-1}$
$K_{double|dim}$	1
$\text {Raf}_{\text {total}}$	110 nM
low [14-3-3]	100 nM
medium [14-3-3]	1 µM
high [14-3-3]	10 µM

## Numerical Results

**Fig. 2:**
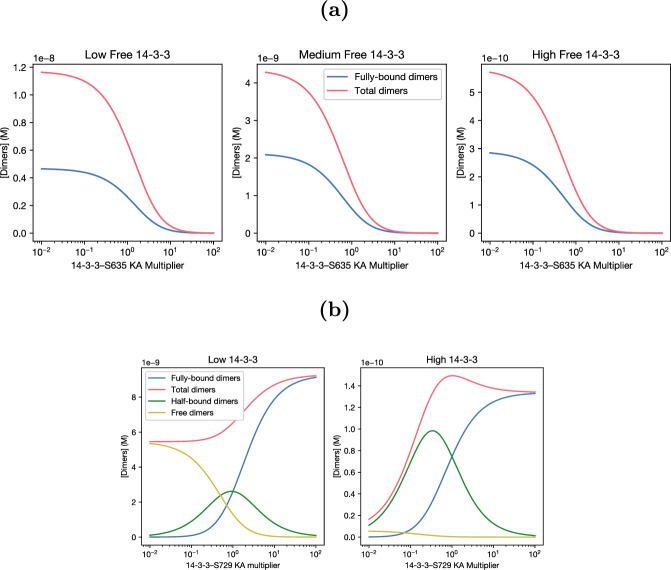
**(a)** At all 14-3-3 levels, increasing the strength of 14-3-3–S365 binding (increasing the $K_A$ multiplier, which increases $K_{365}$) pulls more RAF to the closed conformation and reduces activation. **(b)** Strengthening 14-3-3–S729 binding (increasing the $K_A$ multiplier) moves dimers (and monomers) towards bound complexes, and tends to somewhat increase dimer formation. An equilibrium exists at both strong binding and fully bound RAF and at weak binding and fully unbound RAF

While the analytical solution derived in Section 2 allows us to determine the concentration of RAF states from the binding constants that serve as model parameters, many of those binding constants are effectively unknown, are incompletely defined, or vary between cell types. The total concentration of RAF can be estimated as $\sim$110 nM based on the results in Kulak et al. (2014) and Fujioka et al. (2006), and total concentration of 14-3-3 can be estimated as well (see Table 2). This analysis, which naively gives $\sim$ $100\times$ as much 14-3-3 $\zeta$ and $\eta$ alone (out of the 7 isoforms) as RAF, is complicated by the fact that 14-3-3 plays many other roles throughout the cell (Fu et al. 2000), making it unclear how much is available for interactions with RAF. The values of equilibrium constants are even less clear. For instance, reported binding affinities of 14-3-3 to target sites on RAF range from covalent-like energy differences in Zhang et al. (2021) of hundreds of kcal/mol (from molecular dynamics simulations), to peptide affinities from Ghosh et al. (2015) of $\sim$17 $\upmu$M. To analyze this model given these uncertainties, we conducted a variety of one- and two-variable parameter sweeps, taking as output the total concentration of RAF dimers (as only dimers are competent to signal (Hu et al. 2013)). While this cannot demonstrate universal properties of the system, it does suggest patterns of responses to parameter variations that can be analytically validated.

**Fig. 3:**
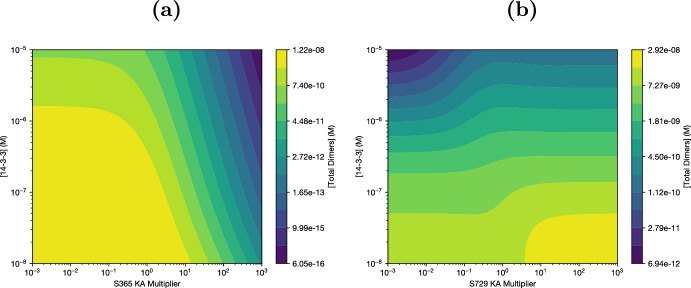
Contours of responses to 14-3-3 and Serine-specific affinity changes. **(a)** Increasing S365 affinity (increasing the $K_A$ multiplier) decreases activation at all 14-3-3 levels, not just the ones sampled in Figure 2a **(b)** The rise in activation corresponding to a change from unbound to bound dimers (see Figure 2b) is visible at various 14-3-3 concentrations. In either state, increasing 14-3-3 tends to decrease dimer levels

A few patterns emerged in our initial 1d parameter sweeps (Figure 2). Unsurprisingly, increasing $K_{365}$ decreased total dimer concentration (see Figure 2a, reduction in total dimers as 14-3-3–S365 $K_A$ ($K_{365}$) increases to the right), as it causes 14-3-3 to more strongly stabilize closed monomers. This is a universal feature of the model, which can be shown by taking the derivative of dimer concentration with respect to $K_{365}$ (see Online Resource 2). Varying bonding constants to S729 ($K_{729}$, $K_{729|O}$, $K_{729|dim}$, $K_{double|dim}$, and $K_{729|365}$) together (Figure 2b) has a more complex effect: increasing the combined affinity (moving right on the graph) decreased unbound dimers and increased bound dimers, moving the system from one activation level to another. At the parameter values considered in Figure 2b and Figure 3b the bound dimer equilibrium was more active than the free dimer equilibrium, but it’s not clear that that would hold throughout all of parameter space. For instance, they must converge at sufficiently low 14-3-3 that few bound dimers can be formed regardless of affinity. In addition to the effects of binding constant variation, increasing 14-3-3 concentration tends to reduce total dimer concentration, as can be seen by comparing the scales of Figure 2: dimer concentrations along the y axis are about 2 orders lower when 14-3-3 is increased from 100 nM to 10 µM.

The parameter sweeps of Figure 2 are limited by the discrete set of 14-3-3 concentrations considered, so we also simultaneously varied both 14-3-3 concentration and equilibrium constants, generating contour plots of dimer concentration (Figure 3). We see the patterns of response to equilibrium constants familiar from Figure 2: decreased dimerization in response to $K_{365}$ (Figure 3a, moving right towards higher $K_{365}$ at constant [14-3-3]) and 2 equilibria at high and low S729 affinities (Figure 3b, again holding [14-3-3] constant and moving left to right through $K_A$ values). It also becomes increasingly clear that, within the chosen parameter ranges (14-3-3 $K_D$ around 100 nM, double-binding $K_D$ around 1, 14-3-3 concentrations between 100 nM and 10 $\upmu$M), increasing [14-3-3] (moving up on Figure 3) will consistently decrease dimer concentration. This contradicts the transfection results in Mendiratta et al. (2025), suggesting that something about our parameter range is wrong.

**Fig. 4:**
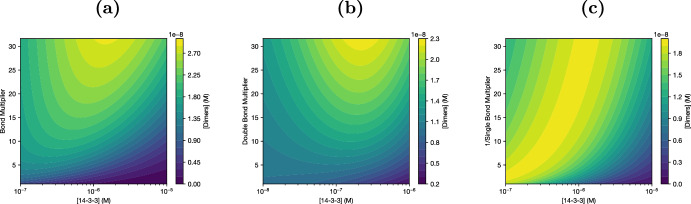
Contour plots of dimers vs [14-3-3] and equilibrium constants. **(a)** As 14-3-3 avidity towards dimers increases and single bonds weaken, the inflection point at which increasing 14-3-3 concentration reduces activation moves right to higher [14-3-3]. **(b)** This effect is not due to the avidity change; modifying $K_{double|dim}$ alone does not move the inflection point. **(c)** Weakening single binding of 14-3-3 to all sites is sufficient to move the inflection point

To understand what causes this response to 14-3-3 concentration, we extend our numerical comparisons to different combinations of affinities in Figure 4. To do this, we define “bond multipliers,” where increasing a multiplier strengthens any binding of a particular type. The single bond multiplier in Figure 4c decreases $K_{365}$, $K_{729}$, $K_{729_O}$, and $K_{729|dim}$ by dividing our default value for each by the same coefficient; moving up on Figure 4c weakens all single binding interactions. The double bond multiplier in Figure 4b is an increase to $K_{double|dim}$; moving up strengthens double binding to dimers. The bond multiplier in Figure 4a combines both effects; moving up both strengthens double binding to dimers and weakens single binding.

Interestingly, as we see in Figure 4b, the inhibitory effect of 14-3-3 on dimer formation is not simply due to weak double binding. Increasing avidity to dimers ($K_{double|dim}$) increases dimer concentration, but it does not significantly affect the response of dimer concentration to 14-3-3: the inflection point where increasing 14-3-3 concentration starts to inhibit dimer formation is largely constant regardless of the double bond multiplier. Instead, to expand the range of 14-3-3 concentration where increasing 14-3-3 increases dimers, it is necessary to reduce the strength of single bonds, either alone (as in Figure 4c) or in combination with double binding (as in Figure 4a). This is not particularly intuitive, and analytic work with the solution was necessary to understand the mechanism leading to this behavior. In addition, as we could only sample a small subset of the possible parameter space, the analytical explanation allows for much stronger general statements about the behavior of the system.

## Analytic Results

### Dimer Derivative

As there exists an explicit solution to the model, it is possible to analytically find the values and derivatives of concentrations of various species. We want to assess the necessary or sufficient conditions for increased 14-3-3 to promote rather than inhibit dimer formation. Our numerical result suggests this is related to the strength of single binding interactions relative to 14-3-3 concentration, but cannot rigorously prove it without a thorough exploration of a large, multidimensional parameter space. Instead, we analytically find the derivative of total dimer concentration with respect to free 14-3-3, and derive the conditions under which it is positive (where an increase in 14-3-3 will increase dimer concentration, and therefore activity of the pathway).

To accomplish this, we found total dimer concentration,

17 $$\begin{aligned} {[}\text {Dimers}] = 2 {[O]}^2 K_{dim} \left( 1 + K_{729|dim}[\text {14-3-3}]\left( 1 + K_{double|dim}\right) \right) , \end{aligned}$$

where [Dimers] = $[Dim_{0}] + [Dim_{1}] + [Dim_{2}]$, and took its derivative with respect to 14-3-3 concentration using Mathematica (see Online Resource 2). The derivative is positive when two conditions hold. The first is that

18 $$\begin{aligned} {[}\text {14-3-3}] < \frac{1 + K_{ai}}{K_{ai}C + K_{729|O}}, \end{aligned}$$

using the definition

19 $$\begin{aligned} C&= K_{365} + K_{729} + K_{729|365}K_{365}, \end{aligned}$$

a recurring parameter in our results which can be interpreted as a total affinity of 14-3-3 to closed Raf. This requirement can be fulfilled as long as the system is not too heavily weighted towards monomers (by requiring sufficiently low $K_{729|O}$ and $C K_{ai}$), and can also be expressed as

20 $$\begin{aligned} K_{ai}\left( [\text {14-3-3}]C - 1\right) + [\text {14-3-3}]K_{729|O} - 1 < 0, \end{aligned}$$

which is necessary for the next derivation.

The second condition is that

21 $$\begin{aligned} B > \frac{-2 (C K_{ai} + K_{729|O})}{K_{ai}([\text {14-3-3}]C - 1) + [\text {14-3-3}]K_{729|O} - 1}, \end{aligned}$$

using the definition

22 $$\begin{aligned} B = K_{729|dim}(1 + K_{double|dim}), \end{aligned}$$

another recurring parameter that may be interpreted as total affinity of 14-3-3 to RAF dimers. Using Equation (20), this is equivalent to

23 $$\begin{aligned} B[\text {14-3-3}]K_{729|O} + B[\text {14-3-3}]CK_{ai} - BK_{ai} - B < -2CK_{ai} - 2K_{729|O}, \end{aligned}$$

which reduces via

24 $$\begin{aligned} B[\text {14-3-3}]K_{729|O} + 2K_{729|O} + B[\text {14-3-3}]CK_{ai} + 2CK_{ai} < BK_{ai} + B \end{aligned}$$

to

25 $$\begin{aligned} \left( B[\text {14-3-3}] + 2\right) \left( CK_{ai} + K_{729|O}\right) < B \left( 1 + K_{ai}\right) . \end{aligned}$$

As total 14-3-3 increases monotonically with free 14-3-3, these conditions also describe when the derivative of dimer concentration with respect to total 14-3-3 is positive. Importantly, these results are valid for all possible parameter values, unlike the parameter sweeps above (Section 3) and the simplifications below (Sections 4.2 and 4.3).

It is clear from Equation (25) that increasing 14-3-3 affinity for dimers (increasing $B$) is not sufficient for higher [14-3-3] to increase dimer concentration. To ensure the inequality holds, there must be either low free 14-3-3 or low affinity for single bonds to monomers ($C$ and $K_{729|O}$). This replicates and generalizes our numerical result in Figure 4. As the total cellular concentration of 14-3-3 is much higher than that of RAF (Kulak et al. 2014), weaker single binding is the more plausible mechanism. In addition, Equation (25) requires that monomer avidity ($K_{729|365}$, as part of $C$) is not too high, or that it is balanced by low single affinities for monomers ($K_{365}, K_{729}$).

### Constant Single Binding

While the derivation of Equation (25) explains our numerical results in Figure 4, its practical implications are harder to read out. Below, we clarify the implications of Equations (25) and (20) for RAF–14-3-3 affinity and avidity, through the use of simplifying assumptions and example values for binding constants.

A useful simplification to understand these results is to hold all single binding constants ($K_{365}$, $K_{729}$, $K_{729|O}$, $K_{729|dim}$) equal, with their value set to $K_{single}$. If you expand Equation (18) using

26 $$\begin{aligned} B&= (1+K_{ai}) K_{single}, \end{aligned}$$

27 $$\begin{aligned} C&= (2 + K_{729|365}) K_{single} \end{aligned}$$

it gives

28 $$\begin{aligned} {[}\text {14-3-3}] K_{single} < \frac{1 + K_{ai}}{K_{ai}(2 + K_{729|365}) + 1}. \end{aligned}$$

If this is stated in $K_D$ terms ($K_{D,single} = 1/K_{single}$), we find

29 $$\begin{aligned} K_{D,single} > [\text {14-3-3}] \left( 1 + K_{ai} \frac{1 + K_{729|365}}{1 + K_{ai}}\right) . \end{aligned}$$

A similar substitution in Equation (25) gives the somewhat unwieldy

30 $$\begin{aligned} (K_{single}[\text {14-3-3}](1 + K_{double|dim}) + 2)(2K_{ai} + K_{ai}K_{729|365} + 1) < (1 + K_{double|dim})(1 + K_{ai}), \end{aligned}$$

which is linear in $[\text {14-3-3}]K_{single}$, and therefore gives another condition where $K_{D,single}$ is a rational expression multiplied by [14-3-3]. To ensure the result for $[\text {14-3-3}]K_{single}$ is positive after the subtraction of the +2 term, a second condition is required:

31 $$\begin{aligned} K_{double|dim} (1 + K_{ai}) > 2 K_{ai} K_{729|365} + 3 K_{ai} + 1, \end{aligned}$$

which serves as a lower bound for $K_{double|dim}$. Overall, we find that $K_{D,single}$ has a lower bound of the greater of two rational expressions multiplied by [14-3-3], and $K_{double|dim}$ must at least exceed a linear expression in $K_{729|365}$.

For a somewhat more concrete example of these bounds, consider the case of $K_{ai} = 1$. Equation (25) then implies

32 $$\begin{aligned} K_{double|dim} > K_{729|365} + 2 \end{aligned}$$

via Equation (31) and

33 $$\begin{aligned} K_{D,single} > [\text {14-3-3}] \frac{(3 + K_{729|365})(1 + K_{double|dim})}{2(K_{double|dim} - K_{729|365} - 2)} \end{aligned}$$

via Equation (30), while Equation (29) reduces to

34 $$\begin{aligned} K_{D,single} > [\text {14-3-3}] \left( \frac{3 + K_{729|365}}{2}\right) . \end{aligned}$$

If we additionally set $K_{729|365} = 1$, this becomes

35 $$\begin{aligned} K_{double|dim}&> 3, \end{aligned}$$

36 $$\begin{aligned} K_{D,single}&> 2 [\text {14-3-3}] \cdot \text {max}(1, \frac{K_{double|dim} + 1}{K_{double|dim} - 3}). \end{aligned}$$

We can justify the initial assumptions of equal single binding constants (and therefore the reasoning leading to Equations (29) and (31)) as approximately accurate since they are all the binding of 14-3-3 to its standard motif; this isn’t strictly accurate but only limited measurements are available, and those often use peptides of the binding motif (e.g. Ghosh et al. (2015)) and therefore also neglect the spatial/large-scale structural differences that could help differentiate the binding locations. The approximations of $K_{ai}$ and $K_{729|365}$ as 1 cannot similarly be justified; these are just examples of the types of relations that exist for $K_{double|dim}$ and $K_{single}$.

Equation (29) suggests that in our constant single binding approximation, $K_{D,single}$ is at least as high as and may be significantly higher than [14-3-3], making single binding fairly weak regardless of the available 14-3-3 concentration. In a system without double binding, the ratio of $K_{single}$/[14-3-3] is by definition equal to the ratio of [free Raf]/[bound Raf]. If the single binding $K_D$ values and 14-3-3 concentration are comparable or the binding constant is greater (e.g., Equations (29), (34) and (36)), then much of the RAF would not be bound to 14-3-3 if not for double binding/avidity. A $K_{double|dim}$ substantially greater than 1, as we see in cases like Equation (32), can then substantially pull this system towards RAF–14-3-3 binding (as could a $K_{729|365} > 1$, which would also imply a larger $K_{double|dim}$ by Equation (31)).

### Double-Binding Limit

If we now consider single binding to be weak, can we use that to make a simpler model of RAF–14-3-3 interactions? Not only is this possible, but it already comprises a component of the drug interaction model in Mendiratta et al. (2025).

To fulfill the conditions 25 and 20, which are required for the observed behavior that RAF dimerization increases as a result of overexpression of 14-3-3, it is necessary for RAF-14-3-3 binding at any single phosphosite to be relatively weak, but significant avidity at the second binding site is still permitted. To simplify the model, consider the limit in which only double binding can occur. At this limit,

37 $$\begin{aligned} K_{729}, K_{365}, K_{729|O}, K_{729|dim} \rightarrow 0, \end{aligned}$$

while the products $K_{729|dim} \cdot K_{double|dim}$ and $K_{365} \cdot K_{729|365}$ are held constant. For this derivation, it is more convenient to use $K_D$ notation than $K_A$ notation (where $K_D = 1/K_A$), so let

38 $$\begin{aligned} C \rightarrow K_{365}K_{729|365}&= 1/K_{sm}, \end{aligned}$$

39 $$\begin{aligned} B \rightarrow K_{729|dim}K_{double|dim}&= 1/K_{sd}. \end{aligned}$$

From there, condition 25 reduces to

40 $$\begin{aligned} K_{ai} \frac{[\text {14-3-3}]}{K_{sm}(K_{sd} + 2)} < (1 + K_{ai})/K_{sd}, \end{aligned}$$

letting us further show

41 $$\begin{aligned} K_{ai} ([\text {14-3-3}] + 2 K_{sd})&< K_{sm} \left( 1 + K_{ai}\right) , \end{aligned}$$

42 $$\begin{aligned}  [\text {14-3-3}] + 2K_{sd}&< K_{sm} \frac{1 + K_{ai}}{K_{ai}}. \end{aligned}$$

This is equivalent to the model in Mendiratta et al. (2025) when their concentration of RAF inhibitor drugs (the topic of both Mendiratta et al. papers) is set to 0, demonstrating that their model of Raf–14-3-3 interactions is equivalent to ours at the limit where only double binding is significant. Since our model implies this must be at least approximately the case for a positive derivative of dimer concentration with respect to 14-3-3 (see Section 4.2), we find that model to be a good approximation of RAF–14-3-3 interactions.

## Discussion

The regulation of RAF kinase activation is complicated, with multiple steps that involve conformational changes, dimerization, relocalization, post-translational modifications, and binding partners (Lavoie and Therrien 2015). These reactions can lead to completely non-intuitive behaviors, as was demonstrated when pharmaceutical RAF inhibitors were found, in some contexts, to result in RAF activation (Poulikakos et al. 2010). Mathematical models can be a useful tool for studying complex systems, and RAF signaling is an appealing topic for modeling because of the complexity and non-intutive behaviors. However, a detailed incorporation of all RAF isoforms, homodimers and heterodimers, conformational changes, post-translational modifications, and other protein-protein interactions would result in a combinatorial explosion of states, which complicates model-building and parameterization. Our approach, therefore, has been to systematically identify the aspects of RAF signal regulation that modulate critical steps of RAF activation and manifest themselves in the observable non-intuitive phenomenon (Mendiratta and Stites 2023; Mendiratta et al. 2025). By identifying whether and how 14-3-3 proteins modulate RAF signaling, and identifying simplifications that are generally valid, efforts like this are facilitating the development of more complex models.

Given the experimental result that increased concentration of 14-3-3 increases RAF dimerization (Mendiratta et al. 2025), our model suggests that 14-3-3 binding to each individual phosphoserine in RAF is likely to be relatively weak, with a $K_D$ of a similar order to the available cellular concentration of 14-3-3. This may help clear up the highly variable results of previous simulations and experimental measurements of the affinity (Ghosh et al. 2015; Tzivion et al. 1998; Zhang et al. 2021). It also validates the simplifying approximation that 14-3-3 only binds in cases where both phospho-sites are available, which we used in previous RAF modeling (Mendiratta et al. 2025) and may reuse in future work.

The numerical analyses was centered around a set of parameters values that were chosen to be consistent with available experimental characterizations of the pathway. Evaluations of the impacts of specific parameters were limited to one or two dimensions at a time, thus studying how productive RAF signaling is modulated by critically important parameters (e.g., 14-3-3 abundance, relative affinities of the two different 14-3-3 binding sites). These numerical evaluations support our more general results from analytic modeling and also provide visual portrayals of analytical results. However, numerical analyses like these do not imply that these behaviors are universal across all of the parameter space. Such conclusions are sometimes possible analytically, and even then the conclusions are only valid for the given model. Global sensitivity analysis methods can also provide input to how parameters tend to modulate a behavior across a parameter space, but are also dependent upon the model being analyzed.

This system resembles Ercolani and Schiaffino’s model of chelate cooperativity (Ercolani and Schiaffino 2011), similar to avidity but considers different limiting cases. Like our work, they find situations where one binding state will tend to become favored at sufficiently high concentrations due to 1:1 vs 2:1 ratios, specifically that at high enough concentrations of BB the “cyclic” double-binding (comparable to monomers) should be disfavored. (Their AA receptor is analogous to 14-3-3 and their BB ligand is analogous to RAF.) However they don’t consider the high AA receptor case, and since their results are in terms of free ligand concentration they don’t include the effects of receptor concentration on the free ligand. This makes sense when considering a ligand-receptor system, as they did, but does not necessarily generalize to other protein-protein interactions. 14-3-3, however, has a substantially higher concentration than RAF (Kulak et al. 2014, though it’s less clear that available 14-3-3 does), and our work was partly motivated by experiments involving 14-3-3 overexpression. The effect of 14-3-3 concentration on free RAF is necessary for the stoichiometric preference for monomers at high 14-3-3 concentrations which we observe here, and our work goes on to show the conditions under which this stoichiometric pressure becomes dominant.

Many questions remain on RAF–14-3-3 interactions. While this result qualitatively constrains the strength of double- vs single-binding of 14-3-3 to RAF, due to the number of free parameters without reliable experimental measurement we cannot assign any specific value to each affinity without assumptions about other values. In addition, as this is not a complete model of RAF activation, any such value derived from this model would not be a physical equilibrium constant between two strictly defined states, but rather a composite over some collection of states. For instance, the value of $K_{ai}$ in this model implicitly includes the effects of RAS binding (as might the 14-3-3–closed monomer affinities, depending the details of the relationship between RAS–RAF binding and RAF–14-3-3 binding (Martinez Fiesco et al. 2022)).

There is also considerable work to be done in RAF modeling more generally. We know of a wide variety of processes involved in RAF activation, including dimerization (and associated internal phosphorylation events), phosphorylation and dephosphorylation of S365 (the latter via the recently-imaged PP1C-SHOC2-MRAS complex (Bonsor et al. 2022; Hauseman et al. 2022; Kwon et al. 2022; Liau et al. 2022)), conformational changes, and RAS binding. The development of larger scale models that include additional steps of RAF regulation and additional binding partners is limited in part by uncertainties in mechanisms of RAF regulation. As models become more complex, numerical results become increasingly important, and the fact that many parameters have not been measured adds additional complexity to developing such models. We believe that focused studies like this that can facilitate the development of larger scope models both by justifying simplifications and by constraining which portions of parameter space better correspond with empirical observations.

## Supplementary Information

Online Resource 1 contains the Python files and Jupyter notebooks used for the numerical analysis in Section 3. Online Resource 2, a Mathematica notebook, includes calculation of the conditions under which various derivatives are positive or negative, notably Equations (20) and (25).

## Data Availability

The Mathematica and Python notebooks used to perform the analytic and numeric studies in this work are included as supplemental information, and are available at https://github.com/pcarlip/14-3-3-final. Protein abundance data was derived from Kulak et al. (2014) and Fujioka et al. (2006) (see Table 2).
